# Geographic isolation and larval dispersal shape seascape genetic patterns differently according to spatial scale

**DOI:** 10.1111/eva.12638

**Published:** 2018-06-05

**Authors:** Alicia Dalongeville, Marco Andrello, David Mouillot, Stéphane Lobreaux, Marie‐Josée Fortin, Frida Lasram, Jonathan Belmaker, Delphine Rocklin, Stéphanie Manel

**Affiliations:** ^1^ EPHE, Biogéographie et Ecologie des Vertébrés CEFE, UMR 5175, CNRS PSL Research University Université de Montpellier Université Paul‐Valéry Montpellier Montpellier France; ^2^ MARBEC, UMR 9190, CNRS, IRD Université Montpellier – Ifremer Montpellier France; ^3^ Laboratoire d'Ecologie Alpine UMR‐CNRS 5553 Université Joseph Fourier Grenoble France; ^4^ Department of Ecology and Evolutionary Biology University of Toronto Toronto Ontario Canada; ^5^ Laboratoire d'Océanologie et de Géosciences UMR 8187 LOG CNRS Université du Littoral Côte d'Opale Wimereux France; ^6^ Department of Zoology and the Steinhardt Museum of Natural History Tel Aviv University Tel Aviv Israel; ^7^ Faculty of Humanities and Social Sciences Department of Geography Memorial University of Newfoundland St John's NL Canada

**Keywords:** connectivity, ecological genetics, marine fish, Mediterranean Sea, *Mullus surmuletus*, seascape genetics, single nucleotide polymorphism

## Abstract

Genetic variation, as a basis of evolutionary change, allows species to adapt and persist in different climates and environments. Yet, a comprehensive assessment of the drivers of genetic variation at different spatial scales is still missing in marine ecosystems. Here, we investigated the influence of environment, geographic isolation, and larval dispersal on the variation in allele frequencies, using an extensive spatial sampling (47 locations) of the striped red mullet (*Mullus surmuletus*) in the Mediterranean Sea. Univariate multiple regressions were used to test the influence of environment (salinity and temperature), geographic isolation, and larval dispersal on single nucleotide polymorphism (SNP) allele frequencies. We used Moran's eigenvector maps (db‐MEMs) and asymmetric eigenvector maps (AEMs) to decompose geographic and dispersal distances in predictors representing different spatial scales. We found that salinity and temperature had only a weak effect on the variation in allele frequencies. Our results revealed the predominance of geographic isolation to explain variation in allele frequencies at large spatial scale (>1,000 km), while larval dispersal was the major predictor at smaller spatial scale (<1,000 km). Our findings stress the importance of including spatial scales to understand the drivers of spatial genetic variation. We suggest that larval dispersal allows to maintain gene flows at small to intermediate scale, while at broad scale, genetic variation may be mostly shaped by adult mobility, demographic history, or multigenerational stepping‐stone dispersal. These findings bring out important spatial scale considerations to account for in the design of a protected area network that would efficiently enhance protection and persistence capacity of marine species.

## 1 INTRODUCTION

Genetic variation is both the outcome and the basis of evolutionary change (Kokko et al., 2017), allowing species to adapt to novel environmental conditions and persist in different climates and environments. Describing the spatial patterns of genetic variation can help to better understand the underlying processes of evolution and is one of the main focuses of landscape genetics (Manel, Schwartz, Luikart, & Taberlet, 2003). Studying the spatial distribution of genetic variation requires both characterizing the genetic structure of populations and detecting the variables influencing its spatial organization.

Geographic isolation, barriers to gene flow, and environmental or landscape features can generate spatial genetic patterns such as isolation by distance (IBD; Kimura & Weiss, 1964), isolation by resistance (IBR), or adaptive genetic isolation (Nosil, Egan, & Funk, 2008). Barriers and resistance to movement are directly related to dispersal of individuals at various life stages. Yet, in marine landscapes (i.e., seascapes), dispersal of most organisms occurs during the pelagic larval stage and depends on oceanic currents that can transport propagules over large distances (White et al., 2010). Larval dispersal is then expected to influence seascape genetic variations, and the resulting genetic patterns often differ from the classical theory of isolation by distance (Selkoe et al., 2016). Hence, a comprehensive assessment of the drivers of spatial genetic variation in marine populations needs to include in a same framework environmental heterogeneity, geographic isolation, and larval dispersal (D'Aloia, Bogdanowicz, Harrison, & Buston, 2014; Selkoe et al., 2016). To this aim, large‐scale datasets and novel analytical methods are required (Manel & Holderegger, 2013).

White et al. (2010) were among the first to show that larval dispersal was a better driver of genetic structure than geography measured as the mere Euclidean distance. Selkoe et al. (2010) moreover demonstrated the importance of considering other ecological and environmental factors to explain seascape genetic patterns. In their study, they showed that habitat (i.e., kelp coverage) was the main variable shaping genetic structure for three fish species*,* thus demonstrating that beyond larval dispersal, the survival capacity within a given habitat also affects gene flow. Seascape genetic patterns can also be shaped by other environmental variables such as temperature or salinity through isolation by adaptation (Nosil, Funk, & Ortiz‐Barrientos, 2009). Environmental gradients may influence both adaptive and neutral genetic variations if the movement of individuals is limited by a strong ecological selection against immigrants (Nosil et al., 2009). Historical events such as demographic fluctuations and colonization are also expected to influence spatial genetic variation (Lowe & Allendorf, 2010). It is now well established that several processes structure genetic variation, but there is still much to be learned about the drivers of seascape genetic patterns (Selkoe et al., 2016). In particular, the relative importance and the spatial scale associated with these drivers are rarely discussed in seascape studies (Riginos, Crandall, Liggins, Bongaerts, & Treml, 2016).

Here, we defined three spatial scales for which we expect different processes to affect fish spatial genetic variation (Jombart, Dray, & Dufour, 2009; Wagner & Fortin, 2013): (i) broad scale where we assume a preponderant effect of colonization, migration, or isolation by adaptation; (ii) intermediate scale at which larval dispersal and adult dispersal are expected to occur; and (iii) local scale, expected to be dominated by processes such as local retention of larvae and local adaptation. We thus hypothesize that geographic isolation primarily shapes genetic structure at large scale, while the influence of dispersal and gene flow may become more prevalent when downscaling (Almany et al., 2013; D'Aloia, Bogdanowicz, Majoris, Harrison, & Buston, 2013).

**Figure 1:**
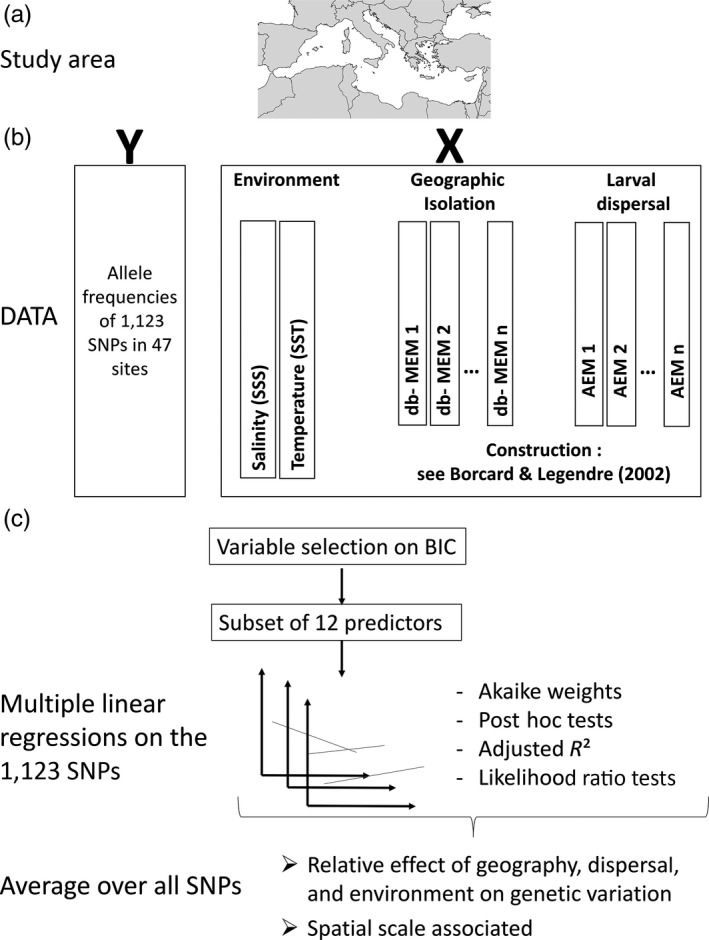
Analytical framework used to test the influence of environment, geographic isolation, and larval dispersal on the genetic variation of *Mullus surmuletus* and their associated spatial scales. (a) The study area, the Mediterranean Sea. (b) The response (SNP allele frequencies) and explanatory variables (environment, geographic isolation, and larval dispersal). Distance‐based Moran's eigenvector maps (db‐MEMs) were used to calculate node‐based predictors of geographic isolation. See Borcard and Legendre (2002) for more detailed explanation on the construction of db‐MEMs. Similarly, asymmetric eigenvector maps (AEMs) were used to calculate node‐based predictors of larval dispersal; construction of AEMs is detailed in Blanchet et al. (2008a). (c) Multiple linear regressions and the Akaike weight used to estimate the relative effect of each type of variable on variation in allele frequencies, and at which spatial scale they are mainly associated

Here, we used the Mediterranean Sea as the archetypal situation where larval dispersal distance and geographic distance may be weakly correlated owing to a very tortuous coastline, several physical barriers (oceanic fronts and gyres), and heterogeneous sea currents (Galarza et al., 2009). This study characterizes the spatial genetic pattern of a heavily exploited species, the striped red mullet (*Mullus surmuletus*). The striped red mullet is a demersal fish species inhabiting coastal shelf ecosystems from 0 to ~100 m depth. This species is widely distributed in the eastern North Atlantic Ocean, from the British Isles in the North to Senegal in the South, including the Mediterranean and Black Sea (Tserpes et al., 2002). It is among the most economically valuable species in the Mediterranean Sea. Spawning occurs from May to July and produces larvae with a pelagic stage lasting for approximately 25–35 days (Macpherson & Raventos, 2006). A change in distribution of the species toward deeper waters during spring, just before the spawning season, has been shown by Machias, Somarakis, and Tsimenides (1998). In most demersal fish species, larval dispersal is presumed to play an important role in shaping the genetic variation of *M. surmuletus*. However, the magnitude of its effect relative to other processes and the spatial scales of its action remain poorly known. Hence, we tested the influence of environmental variables, geographic isolation, and larval dispersal to explain the genetic variation of *M. surmuletus* (Figure 1).

## 2 MATERIALS AND METHODS

### 2.1 Study area, sampling design, and environmental variables

**Figure 2:**
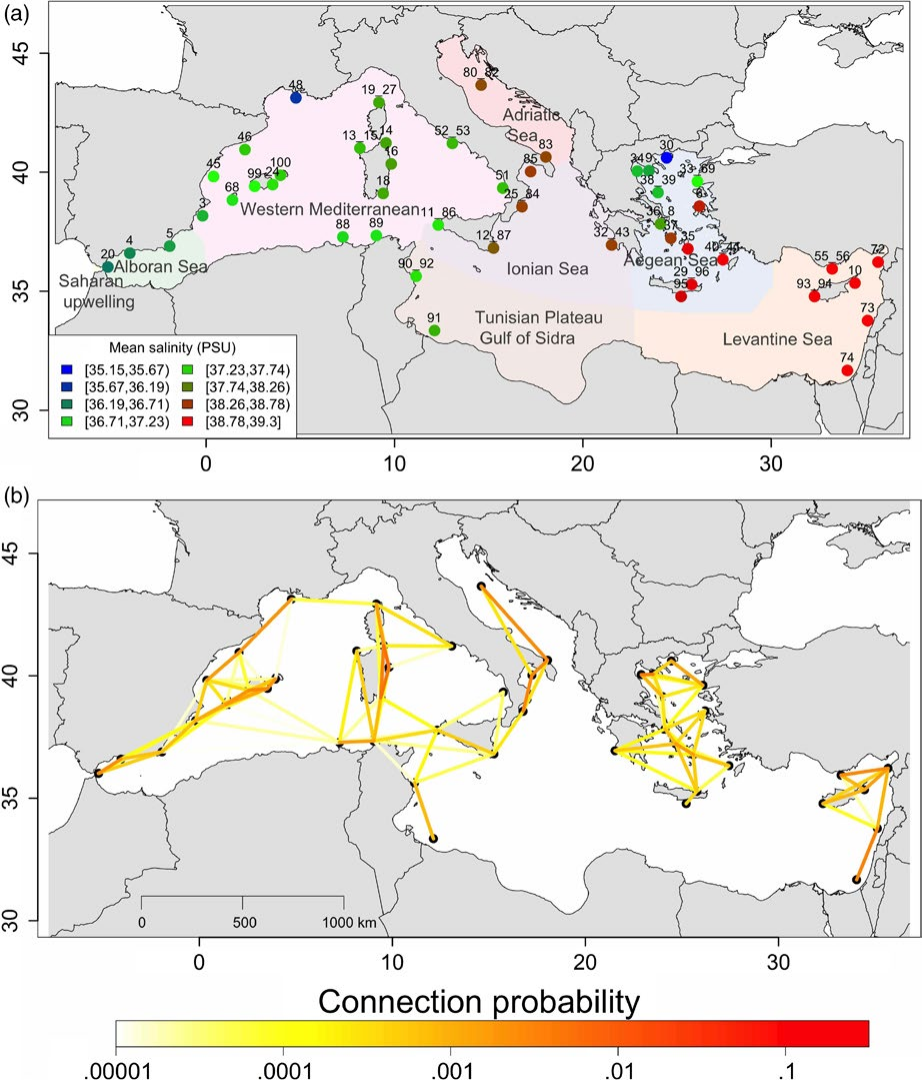
Maps of sampling and larval dispersal of *Mullus surmuletus*. (a) Map of the Mediterranean basin showing the location of the 47 sampling sites in the eight marine ecoregions of the world: Adriatic Sea (red; two sites), Aegean Sea (blue; 12 sites), Alboran Sea (green; three sites), Ionian Sea (purple; five sites), Levantine Sea (orange; six sites), Saharan Upwelling (yellow; one site), Tunisian Plateau/Gulf of Sidra (brown; two sites), and western Mediterranean (pink; 16 sites). The color gradient indicates the mean sea surface salinity at each site. (b) Map of the pairwise probabilities of receiving larvae between sites. The color gradient indicates the larval dispersal probabilities between pairs of sites. Although larval dispersal between two sites is directional, for simplicity only the stronger connection probability is represented in this figure (i.e., the fraction of larvae originating in MPA *j* that ended up in MPA *i*). The larval dispersal probabilities identify three isolated groups

The study area covers the entire Mediterranean coastline, including islands. A total of 727 adults of *M. surmuletus* were collected between April and November 2014 in 47 sites embracing the whole range of the Mediterranean Sea at a fine resolution (≈100 km between sampling locations on average) (Figure 2a; Table S1). The mean distance between our sampling sites was about 100 km. Specimens were obtained from small‐scale fishery landings in each site: The local origin of the samples was confirmed by the fishermen. Fish samples consisted of fin clips of pectoral and caudal fins conserved in 96% ethanol prior to storage at 4°C.

Sea surface temperature (SST) and sea surface salinity (SSS) were computed in each site from the oceanographic model NEMOMED8 (Somot, Sevault, & Deque, 2006), which has a spatial resolution of 1/8° (Albouy et al., 2015) from the period 1990–2013. The daily data were averaged over the whole period for both SST and SSS to infer the mean values. The SSS values of each site are shown in Figure 2a and the SST values in Figure S1.

### 2.2 Genetic data and analyses

Extraction of genomic DNA was undertaken using the DNeasy Blood & Tissue Kit (Qiagen) according to the manufacturer's protocol. DNA quality was assessed by running 3 μl of each DNA sample on 1% agarose gels. DNA concentration was determined using NanoDrop 8000. A total of 558 DNA samples that fulfilled the quality and quantity criteria (DNA of high molecular weight, with at least 40 μl of DNA available at a concentration >10 ng/μl) were then sequenced. Individuals were genotyped using genotyping‐by‐sequencing technique (GBS) based on the use of restriction enzyme digestion to sequence a reduced portion of the genome (Elshire et al., 2011). Libraries were constructed using restriction enzyme ApeKI (recognition site: GCWGC) following a protocol modified from Elshire et al. (2011). Six 96‐plex GBS libraries were prepared and sequenced at the Institute of Genomic Diversity at Cornell University using the Illumina HiSeq 2500 (100 bp, single‐end reads). Each library was sequenced on a separate HiSeq flowcell lane.

Raw read sequences were filtered according to quality base calling, removing sequences with average Phred quality below 25. Trimming was performed to remove low‐quality bases at the extremities of the reads (Phred quality below 20). Sequences shorter than 60 bp were discarded from the dataset. For each sequenced library, the number of raw reads and filtered data are provided in Table S1. SNP calling per pool was performed using the Tassel 3.0 Universal Network‐Enabled Analysis Kit (UNEAK; Lu et al., 2013). We refiltered the individual raw sequences with Stacks software (Catchen, Amores, Hohenlohe, Cresko, & Postlethwait, 2011), which confirmed the low coverage and high rate of missing data for individual genotype calling. To overcome this bias, we considered pools of individuals sampled at the same site rather than the individual genotype information. This strategy produced a dataset of 47 “pools” (i.e., sampling sites) containing between nine and eighteen individuals (Table S1), and whose sequence coverage was >10× at 1153 SNPs. The parameters used in both UNEAK and Stacks are detailed in Appendix S1. We verified that the uneven sample sizes did not influence the estimations of allele frequencies in the 47 pools (see Figure S2).

To remove the potential confounding effect of SNPs under selection, we eliminated candidate SNPs (see Dalongeville, Benestan, Mouillot, Lobreaux, & Manel, 2018). Six genome scan methods were applied to detect selection signal, and the sequences of all the outlier SNPs were blasted on known annotated genes in the NCBI's nr database (NCBI Resource Coordinators, 2017). The blast analysis identified 30 sequences corresponding to genes. We removed the 30 SNPs corresponding to these sequences to produce the final allele frequencies at 1123 SNPs in 47 sites for subsequent analyses.

Genetic differentiation between the pairs of 47 sites was quantified by the Wright's pairwise *F*_ST_, calculated using the R package “polysat” (Clark & Jasieniuk, 2011). To test for isolation by distance (IBD), we performed a Mantel test between pairwise *F*_ST_ computed from allele frequencies and marine geographic distances, computed as least‐cost path distances with infinite resistance values assigned to landmasses. The Mantel test was performed using the R package “*vegan”* (Oksanen et al., 2016) and its significance using 10,000 randomizations.

### 2.3 Spatial and dispersal distances

Geographic isolation was measured as Euclidian distances between pair of sites: We converted degrees latitude and longitude to Cartesian coordinates, and computed the Euclidian distance matrix.

Larval dispersal was quantified using the biophysical model of Andrello, Jacobi, Manel, Thuiller, and Mouillot (2015), which estimates the probability of larval connection between each pair of sites. The model was parameterized with a pelagic larval duration of 30 days and release of larvae every 3 days from the May 1 until the May 28, which corresponds to the spawning season of the species in Mediterranean waters. Larvae were released in 1/10th‐degree cells, and probabilities of dispersal were averaged over the cells belonging to each of the 47 sites, providing a 47 × 47 larval connectivity matrix (Figure 2b).

### 2.4 Conversion to site‐based variables

Pairwise geographic and dispersal distances were converted to site‐based variables used as explanatory variables in the multiple regressions. To do so, we used distance‐based Moran's eigenvector maps (db‐MEMs; Dray, Legendre, & Peres‐Neto, 2006), representing geographic isolation of each site (Figure 1b). The Euclidian distances were decomposed into a set of independent spatial variables (db‐MEMs) using a PCA and eigenvector computations. We used a truncation distance of 4 times the largest distance between sites, as advised in Borcard and Legendre (2002). More details on the calculations of db‐MEMs are provided in Appendix S1. These eigenvectors were used as spatial predictors in regression analyses. db‐MEM analysis was performed using the packages “vegan” (Oksanen et al., 2016), “PCNM” (Legendre, Borcard, Blanchet, & Dray, 2012), “boot” (Canty & Ripley, 2015), and “packfor” (Dray, Legendre, & Blanchet, 2013) in R v. 3.2.3 (R Core Team 2015).

To account for the directionality of larval dispersal, we applied asymmetric eigenvector maps (AEMs) modeling (Blanchet, Legendre, & Borcard, 2008a; Figure 1b). From the pairwise larval connectivity matrix, we constructed a connection diagram linking the 47 sites to one another according to the larval dispersal probability matrix (i.e., an edge is present if the probability of connection between two sites is different from 0), following the procedure described in Blanchet, Legendre, and Borcard (2008b). This diagram was converted into a sites‐by‐edges matrix *E* in which each row is a site and each column is an edge (connection). The sites‐by‐edges matrix *E* was filled with 0s and 1s representing the absence or presence of the various edges linking each site to a hypothetical site at the root of the connection diagram (here located in the Atlantic, as the surface water flow is coming from the Atlantic into the Mediterranean).

To construct the AEMs, a single value decomposition (SVD) was then computed on the column centered *E* matrix (Legendre & Legendre, 2012), and the resulting matrix of site coordinates on the SVD axes was used as the new matrix of explanatory variables reflecting larval dispersal. AEMs were computed using the R package “aem” (Blanchet, Legendre, & Gauthier, 2015).

**Figure 3:**
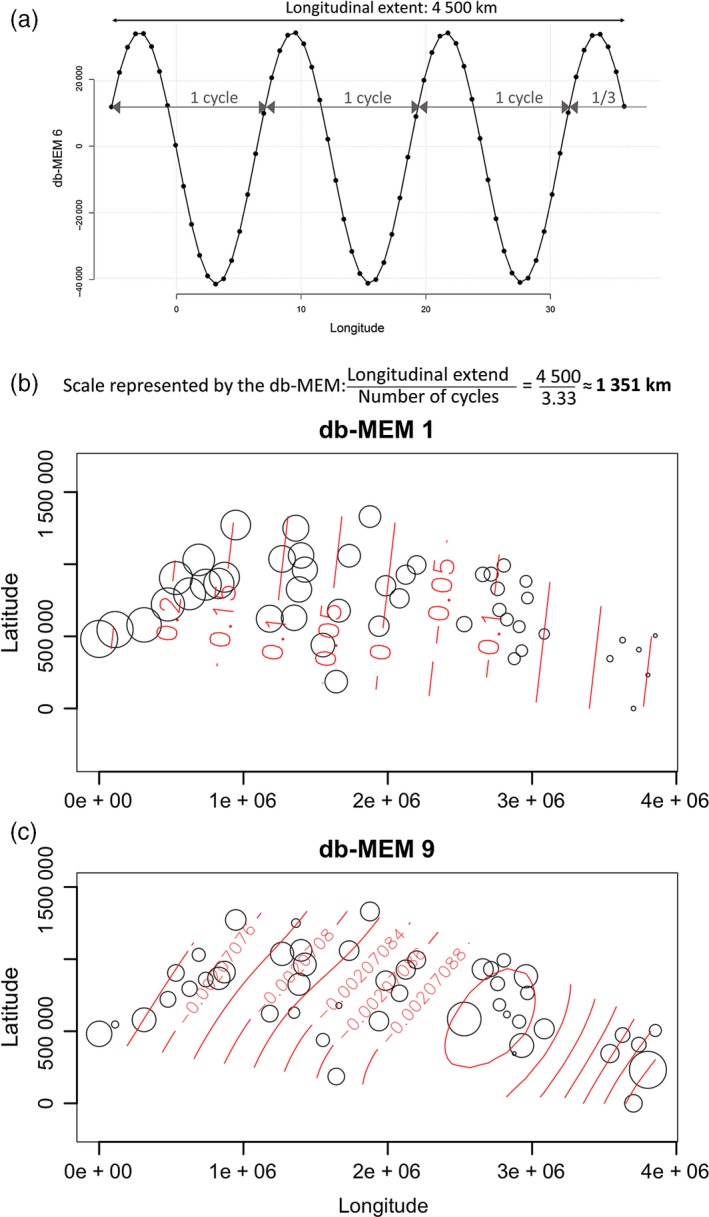
Coordinates of the sampling sites on db‐MEMs and the spatial scale they represent. (a) Theoretical system illustrating how to calculate the scale represented by db‐MEMs. The system covers the same longitudinal extent as our study area (4,500 km) and is constructed from 63 sampling points spaced by an equal distance (the smallest distance between our sampling sites—47 km). The graph presents the coordinates of these sites on the sixth theoretical db‐MEM. The scale represented by the vector is the ratio of the longitudinal extent to the number of cycles of the sinusoid. (b,c) Bubble plots illustrating the db‐MEMs 1 (b) and 9 (c) corresponding to the potential spatial scales of variability based on the geographic distances among sites. The size of the bubble reflects the coordinate of the site on the db‐MEM. Contour lines (in red) show the db‐MEM scores. The bubble plots have been created using the “*ordisurf”* function of the “*vegan”* R package version 2.4‐6 (Oksanen et al., 2016)

The db‐MEMs and AEMs associated with the largest eigenvalues represent the large‐scale spatial structure in the data, while those associated with smaller eigenvalues reflect finer‐scale variations (Borcard & Legendre, 2002; Dray et al., 2006). The spatial scale represented by each db‐MEM can be broadly estimated using the method described in Figure 3a. For example, db‐MEM 1 describes spatial relationship among the study sites at about 4,500 km (Figure 3b), and db‐MEM 9 describes the spatial relationship at about 600 km (Figure 3c).

### 2.5 Data analysis

#### 2.5.1 Variable selection

To reduce the number of predictors used in the regressions, we first reduced the number of variable within each type of spatial predictors (db‐MEMs and AEMs). As we have a high number of SNP, we used the loading scores of the 47 sites on the first axis of a PCA computed on all SNP allele frequencies as a response variable. The spatial predictors were selected using both forward selection and backward selection based on the Bayesian information criterion (BIC) using the MASS package version 7.3.45 (Venables & Ripley, 2002). This step allowed us to build a set of seven db‐MEMs and six AEMs. Then, we calculated Pearson's correlation coefficient between each pair of explanatory variables (db‐MEMs, AEMs, SST, and SSS) and removed one of the variables when the correlation was higher than 0.5. Three variables were eliminated, leading to a final dataset of twelve uncorrelated predictors: two environmental variables (mean SSS and SST), five db‐MEMs, and five AEMs. Prior to further analyses, the 12 explanatory variables were standardized between 0 and 1.

#### 2.5.2 Regression analyses

We performed multiple linear regressions between the allele frequency at each SNP and the twelve explanatory variables accounting for the respective influence of environment, geographic isolation, and larval dispersal (Figure 1c). The explanatory power of a given model, the Akaike weights (AIC_w_; Burnham & Anderson, 2002)*,* was calculated from all possible models (i.e., using all possible combinations of the 12 explanatory variables). The relative importance of each predictor variable at explaining allele frequencies of each SNP was estimated by summing the AIC_w_ values across all models that included that variable and averaged over the 1123 SNPs to obtain their global contribution (ω). Analyses were conducted using the R package MuMIn version 1.15.6 (Barton, 2016). We tested the significance of the differences in mean ω among the twelve predictors using a Kruskal–Wallis test (Hollander & Wolfe, 1973), followed by a post hoc Dunn test (Dunn, 1961) to check the significance of pairwise differences, using the R package “*dunn.test”* version 1.3.5.

To determine whether geographic isolation or larval dispersal had a larger influence on spatial distribution of allele frequencies, we used the likelihood ratio (LR) tests. This test compares the full model (with both db‐MEMs and AEMs) to nested models that consider only geographic isolation (five db‐MEMs) or only larval dispersal (five AEMs). The LR test was performed only on the best models (AIC_min_) for each SNP with at least one significant explanatory variable (693 SNPs). The effect of environmental variables was not tested in these models as SST and SSS showed low contribution to the variation in allele frequencies. We computed the adjusted *R*² of the model with AIC_min_ for each SNP and averaged it across SNPs.

## 3 RESULTS

### 3.1 Variations in allele frequencies across the Mediterranean Sea

Pairwise *F*_ST_ ranged from 0.018 to 0.065, with a mean of 0.033, showing weak genetic differentiation between sites (Figure S3), as expected for a mobile marine species (Waples, 1998). The first row of the *F*_ST_ matrix indicates strong pairwise differentiation between Gibraltar (site 20) and all the other sites. The Alboran Sea (sites 3, 4, and 5) also displays slightly stronger *F*_ST_ values with the rest of the Mediterranean Sea. The Mantel test between pairwise *F*_ST_ and marine geographic least‐cost distances was significant (*r*_M_ = 0.30, *p*‐value < .001), suggesting a pattern of isolation by distance in the data (Figure S4).

### 3.2 Drivers of the spatial distribution of allele frequencies

**Figure 4:**
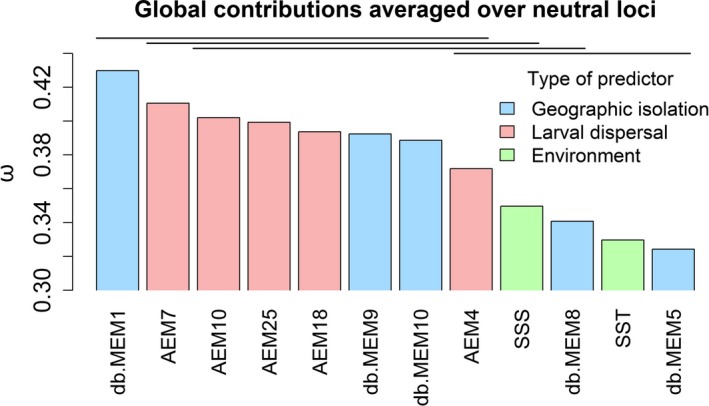
Importance of the explanatory variables on the variation in allele frequencies of *Mullus surmuletus*. Sum of variables’ contributions (ω) over all models including that variable averaged across all SNPs. ω represents the importance of each of the 12 variables to explain variation in allele frequencies. The two environmental variables (mean sea surface temperature—SST and mean sea surface salinity—SSS) are represented in green, the five geographic isolation vectors (db‐MEMs) in blue, and the five larval dispersal vectors (AEMs) in red. Horizontal segments show groups of variables not significantly different according to Dunn's post hoc test

The sums of AIC_w_ of each of the 12 variables calculated from the regression analysis were averaged across all SNPs to obtain their global contribution ω. Averaging the effect of the explanatory variables over all loci informs about general genetic patterns at the scale of the whole genome. The five AEMs (4, 7, 10, 18, and 25) and three db‐MEMs (1, 9, and 10) had ω values significantly higher than the remaining predictors (Figure 4), showing an influence of both geographic isolation and larval dispersal on the variation in *M. surmuletus* allele frequencies. The two environmental variables (SSS and SST) had statistically significantly smaller effect (respectively, 9th and 11th highest ω values; Figure 4).

Considering only the best models for each SNP (AIC_min_), on average larval dispersal and geographic isolation explain, respectively, 11% and 9.66% of the variation in allele frequencies, with no significant difference between the two types of predictors (Table 1). Together, geographic isolation and dispersal explain 19% of the variation in allele frequencies. We calculated the number of time each predictor was selected in the best model across all SNPs (Figure S5). There was no significant difference in the number of selections between the 12 variables.

**Table 1:** Parameters used in multivariate regressions including geographic isolation (db‐MEMs) alone, larval dispersal (AEMs) alone, and both geography and dispersal as explanatory variables of genetic variation (SNPs allele frequencies) of *Mullus surmuletus*

	# SNPs	% of SNPs	Mean adjusted *R*²	Mean AIC	LR test
Null	1123	100	0	−229.6	
Geography	574	51	.096	−231.54	51.47
Dispersal	577	51	.113	−232.03	50.85
Geography + Dispersal	693	62	.187	−235.76	

We considered only the best models for each SNP (AICmin) and only the SNPs for which the best model was better than a model with just an intercept (i.e., null model; ΔAIC > 2; 693 SNPs). The effect of environmental variables was not tested in these models as both variables showed low contribution to the variation in allele frequencies. The table gives an averaged value overall SNPs of adjusted *R*², AIC, and likelihood ratio test comparing, respectively, geographic isolation and dispersal to the full model.

### 3.3 Scale effect on the drivers of allele frequencies

To disentangle the effect of geographic isolation versus larval dispersal, we distinguished three spatial scales: broad, intermediate, and local. The db‐MEMs with the highest global contribution ω represented mostly broad and intermediate spatial scales (db‐MEMs 1, 9, and 10; Figure 4), whereas the most important AEMs described mainly intermediate to local scales (AEMs 4, 7, 10, 18, and 25; Figure 4). db‐MEM 1 described spatial relationship among the study sites at about 4,500 km and suggests three clusters with longitudinal distribution: the western basin and the Adriatic Sea pooled together, the Aegean Sea, and the last cluster made of the six most eastern samples (Figure 3b).

## 4 DISCUSSION

### 4.1 Drivers of genetic variation

To explain the genetic variation of *M. surmuletus*, we took advantage of an extensive (~4,500 km) and fine resolution (~100 km) sampling covering the entire Mediterranean Sea. We show that larval dispersal is the major variable influencing allele frequency variation (mean adjusted *R*² over all SNPs = 11%) at small and intermediate spatial scales (<about 1,000 km), while geographic isolation is the main driver (mean adjusted *R*² over all SNPs = 9.6%) at larger scale (>about 1,000 km). Environmental variables (salinity and temperature) have significantly weaker effects on the variation in allele frequencies (respectively, 9th and 11th highest ω value; Figure 4). The effect of SSS and SST on presumed neutral SNPs could be due to adaptation: If the movement of individuals is limited because of strong ecological selection, adaptation would occur in a form that could impede gene flow via high mortality of immigrants (isolation by adaptation; Nosil et al., 2009). In this case, populations would diverge at both adaptive and neutral loci (Schoville et al., 2012).

Our findings stress the importance of including spatial scale to better interpret the effects of the drivers of genetic variation. The effect of larval dispersal on genetic variation is well ascertained for marine organisms. First for benthic sessile organisms, with adults relatively immobile, larval dispersal is a key process involved in population connectivity (Banks et al., 2007). Banks et al. (2007) showed that fine‐scale genetic structure of the sea urchin (*Centrostephanus rodgersii*) was influenced by sea surface temperature (SST) variability and geography, most likely due to dispersal at the larval stage. A recent study based on SNPs genotyping of the greenlip abalone (*Haliotis laevigata*) showed that its adaptive genetic structure along southern Australian coast has been influenced by environmental heterogeneity. This pattern probably results from adaptation to minimum sea surface temperature and oxygen concentration (Sandoval‐Castillo, Robinson, Hart, Strain, & Beheregaray, 2018). In marine fishes, Schunter et al. (2011), Munguia‐Vega et al. (2014), and Young et al. (2015) showed that fish larval dispersal is a better explanatory factor of genetic variation than geographic distance at spatial scales ranging from 250 km to 4,000 km. Other studies highlighted that, beyond larval dispersal, some environmental factors determine genetic variation of marine fishes. For instance, Selkoe et al. (2010) showed that the main driver of genetic differentiation in *Paralabrax clathratus* is kelp coverage, while Teacher, André, Jonsson, and Merilä (2013) detected an effect of temperature and salinity on the genetic structure of *Clupea harengus*. However, none of these studies have investigated the combined effects of dispersal, geographic isolation, and environment at different spatial scales.

Our analytical framework offers a robust tool to quantify the importance of geographic isolation and larval dispersal in structuring the variation of allele frequencies across spatial scales. We used node (i.e., site)‐based analyses (Wagner & Fortin, 2013), whereas population genetic studies usually use link‐based approaches with genetic distances between pairs of sites. Node‐based approaches allow for robust inferences based on multiple regression techniques and Akaike information criteria (AIC) while avoiding the problems of nonindependency between data arising in link‐based approaches (Legendre, Fortin, & Borcard, 2015). Our analyses rely on the decomposition of directional larval dispersal and geographic isolation into several vectors representing multiple spatial scales, using, respectively, asymmetric eigenvector maps (AEMs) and distance‐based Moran's eigenvector maps (db‐MEMs). This framework is in its early stage of development in landscape genetics (Benestan et al., 2016; Bothwell et al., 2012) and opens new perspectives to interpret the patterns of genetic variation and to understand the processes behind the observed spatial genetic patterns at different scales (Manel & Holderegger, 2013).

### 4.2 Spatial scale associated with these drivers

Our approach based on Moran's eigenvector decomposition allowed us to identify the spatial scales associated with symmetric (db‐MEMs) and asymmetric (AEMs) spatial variables. At our broadest spatial scale—about 4,500 km—the first db‐MEM (db‐MEM 1) has the highest contribution to allele frequency variation (ω), whereas no broad‐scale AEM was selected by the stepwise variable selection procedure. It suggests that geographic isolation is the main driver of variation in allele frequencies at this scale. As no large‐scale AEM was detected as significant, genetic variation at broad scale cannot be explained by passive larval dispersal. At such scale, genetic connectivity could result from processes such as adult mobility, demographic history, or multigenerational stepping stone larval dispersal (D'Aloia et al., 2015) that were not tested in our analyses. Larval dispersal is therefore expected to be better correlated to genetic variation at smaller scales, corresponding to the distances larvae can travel during 30 days.

At intermediate spatial scale, both geography and larval dispersal have an effect on allele frequencies, with AEMs 7 and 10, and db‐MEMs 9 and 10 having the highest contributions (Figure 4). For marine species with a pelagic larval phase, such as *M. surmuletus*, dispersal capacity is primarily determined by pelagic larval duration (PLD), as the possible distance traveled by larvae increases with PLD (Andrello et al., 2013; Selkoe & Toonen, 2011). For example, larvae of the dusky grouper (*Epinephelus marginatus*), which has a similar PLD as *M. surmuletus* (30 days), disperse over distances of about 90 km on average (Andrello et al., 2013). At such scale, oceanic circulation, and thus larval dispersal, is expected to differ from Euclidian geographic distances due to the existence of small‐scale features such as gyres and fronts. In addition to oceanic features, the very tortuous coast of the Mediterranean Sea is likely to limit larval dispersal.

AEM 18 and AEM 25, representing small spatial scales, also display high ω values, whereas no db‐MEM describing small scales was selected by the stepwise variable selection (Figure 4). This result indicates that larval dispersal is more important than geographic isolation to explain local‐scale variations in allele frequencies. Both AEM 18 and AEM 25 show a strong differentiation of one to three populations, located in the Balearic Islands and the Alboran Sea. Interestingly, this corroborates the *F*_ST_ values (Figure S3), which show a slight differentiation of the Alboran Sea with the rest of the Western Mediterranean basin, probably induced by a well‐documented barrier due to oceanic circulation, the Almeria–Oran front (Galarza et al., 2009; Schunter et al., 2011). Processes implicated in the neutral genetic structuration at small scale are related to local oceanic circulation and limited dispersal due to larval retention. Several studies show very high levels of larval retention and self‐recruitment in fish species with long PLD (D'Aloia et al., 2013; Taylor & Hellberg, 2003), which can be due to active swimming of larvae toward the coast or local hydrodynamic retention mechanisms not captured by our dispersal model. Differentiation at local scale can also result from founding (priority) effects that occur when the first dispersers colonizing a new area can influence the success of following settlers (Fraser, Banks, & Waters, 2015).

### 4.3 Limitation of the methods

The power of detecting a significant effect of larval dispersal on the distribution of allele frequencies may be reduced by the loss of information on larval dispersal probabilities in the AEMs. Larval dispersal probabilities were summarized in a node‐by‐edge matrix (*E*), used to build the AEMs. *E* was a binary matrix of connection (i.e.*,* 1 when there is a connection, and 0 when there is not), which ignored the strength of the connections. This representation is required for the computation of AEMs, but it provides coarse information for larval dispersal models, in which dispersal probabilities vary between connections (Figure 2b). Thus, the main effect of larval dispersal on the variation in allele frequencies can be captured by our analyses, but the power of the regressions may be limited by this binary representation.

MEM and AEM analyses are recognized as a relevant way to capture the spatial structure in data (Legendre, Borcard, & Peres‐Neto, 2005) while accounting for different scales of spatial dependence (Borcard & Legendre, 2002). However, MEM and AEM analyses sometimes overestimate the importance of spatial variables when the eigenvectors account for random spatial variations (Gilbert & Bennett, 2010). In our regression analyses, we used the same number of db‐MEMs and AEMs; thus, we do not expect overestimation of geographic versus dispersal variables. However, the importance of environmental predictors compared to that of geographic and dispersal ones could be underestimated in our analyses, especially as environmental variables show some degree of correlation with specific AEMs or db‐MEMs (up to *r*² = .41 between SSS and AEM 7). Thus, we cannot focus our work on the relative importance of environmental versus geographic or dispersal variables, but more on determining which are the spatial scales where geographic isolation and larval dispersal influence the variation in allele frequencies.

## 5 CONCLUSION

Given that genetic variation is closely related to the adaptive and resilience potential of populations (Kokko et al., 2017), the management of fisheries and marine protected areas (MPAs) would benefit from a better understanding of the processes and spatial scales influencing genetic variation of marine species. Larval dispersal is a key ecological process driving population source–sink dynamics and gene flow (Selkoe et al., 2016). Specifically, gene flow and demographic connectivity maintain genetic diversity (Baguette, Blanchet, Legrand, Stevens, & Turlure, 2013), thus promoting population resilience after disturbance (Baguette et al., 2013; Hughes & Stachowicz, 2004). A recent modeling study of Magris et al. (2018) showed the importance of integrating hydrodynamics and larval dispersal in the design of MPA networks to enhance their effectiveness in terms of species persistence. Funk, Mckay, Hohenlohe, and Allendorf (2012) argued that genomic information, both neutral and adaptive, can greatly improve the delineation of conservation units. Sandoval‐Castillo et al. (2018) provided an example of marine genomic and connectivity information being directly used by a government for management and conservation purposes.

We show that larval dispersal influences genetic variation at small and intermediate scales, but not at broad scale where simple geographic distances primarily explain observed allele frequency variation, which may result from other processes such as adult mobility, demographic history, or multigenerational stepping‐stone dispersal. In order to maintain genetic variations in populations of *M. surmuletus*, and more generally in populations of demersal fishes, particular attention should be given to the spacing of MPAs. Indeed, a network of reserves that can be connected by larval dispersal would efficiently protect gene flow between protected areas. Such a network is thus likely to conserve the genetic diversity and adaptive potential of species, and to support renewal of fishery stocks (Olds et al., 2016).

## DATA ARCHIVING STATEMENT

Data for this study are available at the Dryad Digital Repository: https://doi.org/10.5061/dryad.31tk592.

## CONFLICT OF INTEREST

None declared.
