# Supplementary material for: Geographic isolation and larval dispersal shape seascape genetic patterns differently according to spatial scale
Source: Evol Appl. 2018 Jun 5;11(8):1437–47. doi: 10.1111/eva.12638 (PMC6099820; doi:10.1111/eva.12638)
Supplement: Supplementary file 1 [file EVA-11-1437-s001.docx]

**SUPPORTING INFORMATION**

**Appendix S1. Supplementary methods: sequence filtering and SNPs calling**

*SNPs calling using UNEAK*

SNP calling was performed using the Tassel 3.0 Universal Network Enabled Analysis Kit (UNEAK; Lu *et al.*, 2013). UNEAK is a non-reference GBS SNP calling pipeline that has been developed as an extension of the Java program TASSEL (Bradbury *et al.*, 2007). UNEAK proceeds in three steps: (1) the reads are cut to 64bp; (2) these sequences are gathered in tags (*i.e.* identical sequences) for each individual sample; (3) The tags are aligned by pair with a single mismatch. In Uneak plugin UMergeTaxaTagCount, the maximum tag number was set to 10^9^ and the minimum tag for a count to 5 (options –m and –c respectively).

The output file describing the coverage of the alleles for each detected SNP in each individual was used for further filtering of the data. SNPs were first filtered individually to get loci with the best coverage and significant reliability in SNP calling: (a) a minimal mean coverage of 5 among the individuals, (b) a maximal mean coverage of 10 among the individuals, to discard repetitive sequences. The maximum coverage threshold was defined according to distribution of individual coverage in this experiment (mean = 3.11 ± 3.56). Further filtering was performed to remove individuals with an insufficient number of sequences (< 3000 reads). For each SNP, we calculated the ratio of individuals for which the coverage was 0 or 1, and removed SNPs from the dataset when this ratio was higher than 10%.

As the individual coverage of the sequencing was low, we pooled individuals collected in the same site (9 to 18 individuals per pool) to get a more reliable coverage. The reads corresponding to the loci that passed the filters detailed in the previous paragraph were gathered for every individuals in each pool. SNPs were re-filtered to keep a minimal coverage of 10 in each pool with no missing data, which lead to a total of 1153 SNPs. Then, allele counts and allele frequencies per pool have been computed for each of the 1153 SNPs. The filters used for SNPs calling are summarized in Table S2.

*SNPs calling using Stacks*

In order to test whether a different algorithm would improve the dataset, SNPs calling has also been performed with Stacks (Catchen *et al.*, 2011), using the scripts developed in *Stacks workflow* (Normandeau, 2016). Reads were truncated to 80pb, then grouped in tags using the *ustacks* unit with a maximum of three mismatches between sequences (M=3), since an important variability is expected in an abundant fish species such as *M. surmuletus*; and a minimum coverage of 4 (m=4). A catalog de novo was created using the *cstacks* unit, which aligned the reads of a subset of 20 individuals from different geographical locations. The catalog was created with a subset of individuals because the extremely high number of different loci sequenced in all the individuals led to a crash of the program when aligning the sequences (> 3 million loci with 50 individuals analyzed). Individual reads were then aligned on the catalog with the *sstacks* unit, and the *population* unit was used to select loci with an individual coverage higher than 5 (parameter *m*). The output containing individual genotypes has been filtered using the VCFtools software (Danecek *et al.*, 2011) to remove the SNPs with a mean coverage below 8 or above 50. We selected the 250 individuals best sequenced (*i.e.* with the lowest rate of missing data), and removed the SNPs with more than 30% of missing data. Only 50 SNPs passed these filters, which supported the necessity to group individual in pools to infer reliable genotypes for an acceptable number of SNPs. The filters used for SNPs calling are summarized in Table S2.

**References**

Bradbury PJ, Zhang Z, Kroon DE, Casstevens TM, Ramdoss Y, Buckler ES (2007). TASSEL: software for association mapping of complex traits in diverse samples. *Bioinformatics* **23**: 2633–2635.

Catchen JM, Amores A, Hohenlohe P, Cresko W, Postlethwait JH (2011). Stacks: Building and Genotyping Loci De Novo From Short-Read Sequences. *G3 Genes Genomes Genet* **1**: 171–182.

Danecek P, Auton A, Abecasis G, Albers CA, Banks E, DePristo MA, *et al.* (2011). The variant call format and VCFtools. *Bioinformatics* **27**: 2156–2158.

Lu F, Lipka AE, Glaubitz J, Elshire R, Cherney JH, Casler MD, *et al.* (2013). Switchgrass Genomic Diversity, Ploidy, and Evolution: Novel Insights from a Network-Based SNP Discovery Protocol. *PLOS Genet* **9**: e1003215.

Normandeau E (2016). *Command line Stacks Workflow*.

**Supporting Figures**

**Figure S1.** Map of the Mediterranean basin showing the location of the 47 sampling sites. The color gradient indicates the mean sea surface temperature at each site.

**
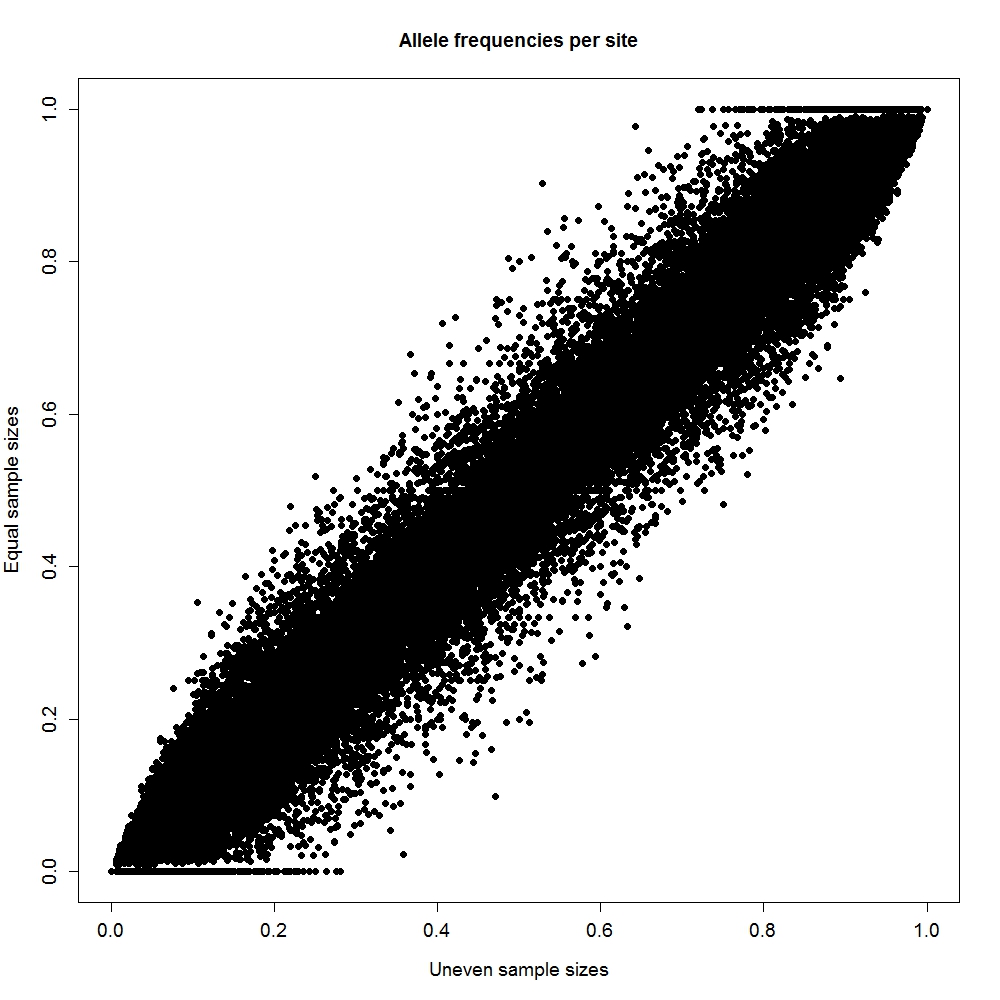
**

**Figure S2.** Bivariate plot of allele frequencies computed from sites with uneven (*x*-axis) and even (*y*-axis) sample sizes. We tested the effect of unequal sample size on allele frequencies estimation by rarefying the data to only nine individuals (i.e. equal sampling size) randomly selected per site, and re-calculating the allele frequencies of every SNPs in each site. For each site, we tested the correlation between these allele frequencies based on even sample sizes of nine individual and the frequencies used in our analyses (computed from uneven sample sizes). Pearson’s correlation coefficients varied between 0.9775 and 1.000 with a mean of 0.9932, and were all highly significant.


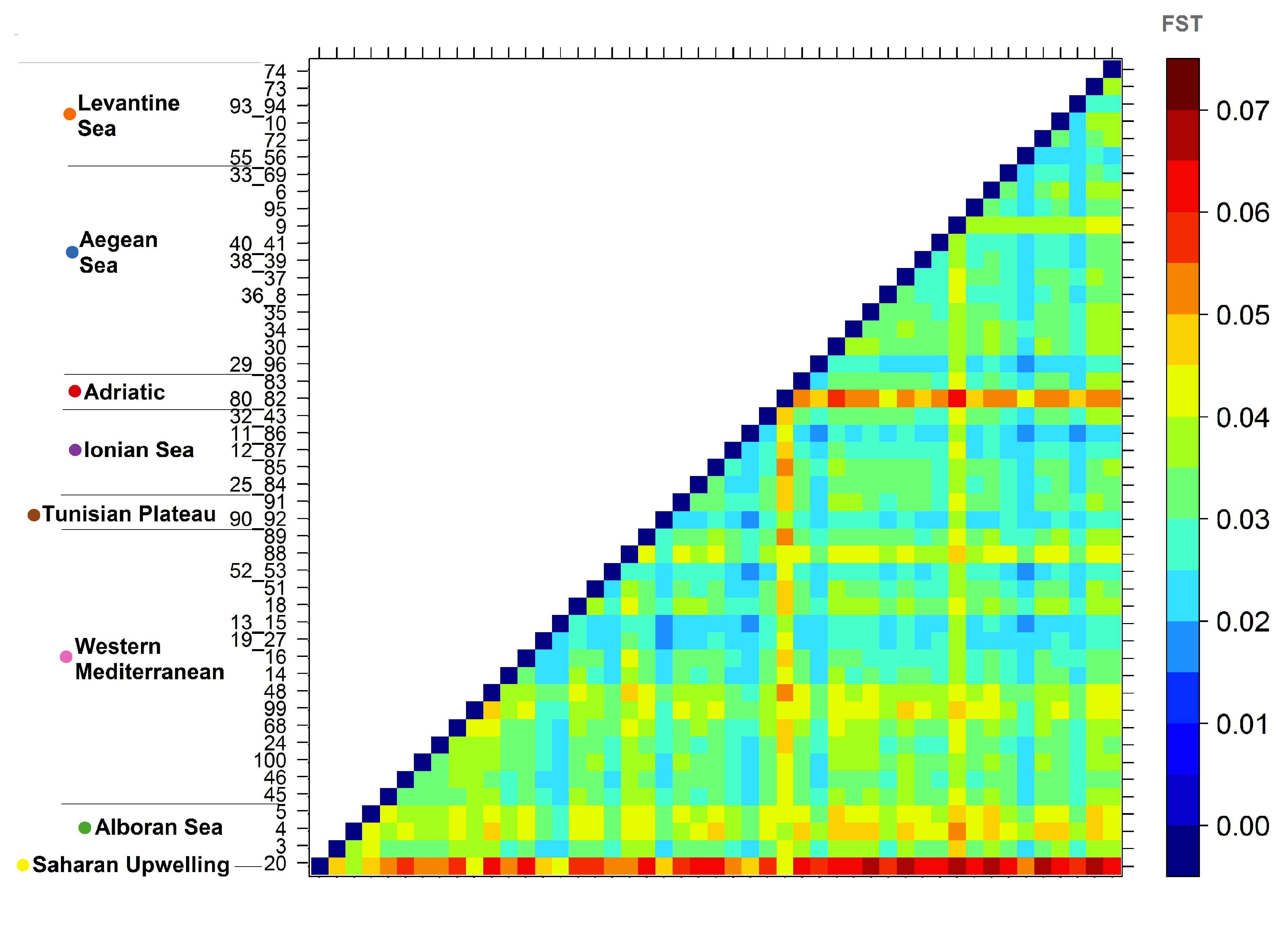


**Figure S3.** Graphical representation of the matrix of pairwise F_ST_ between the 47 sites calculated from SNPs allele frequencies. The location of sites within the eight Mediterranean ecoregions is shown on the left, with their color correspondence to the map of sampling sites (Figure 2a).


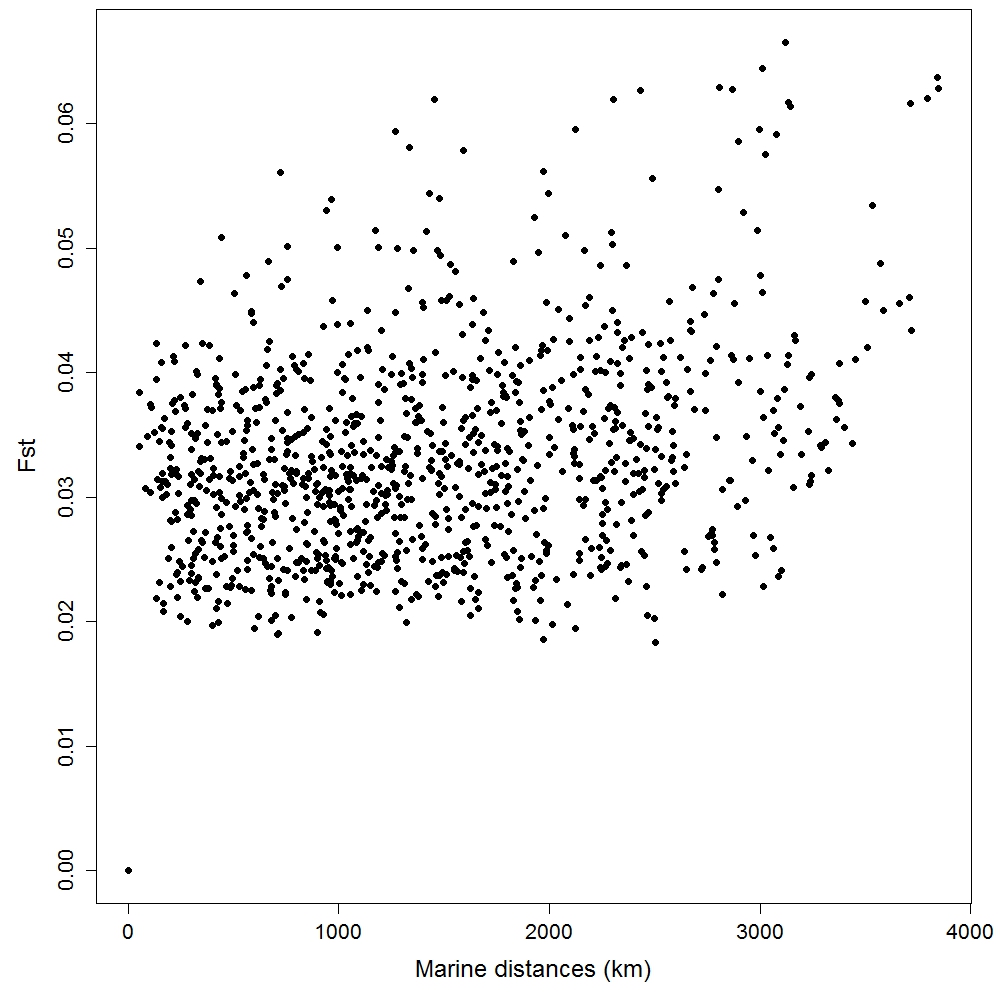


**Figure S4.** Bivariate plot of pairwise F_ST_ vs marine distances between the 47 sites, calculated as least-cost paths with infinite resistance assigned to land masses. The Mantel test between F_ST_ and marine distances was significant with a *r* statistic of 0.30 (*p*-values < 0.001), suggesting Isolation-by-Distance (IBD) in the data.


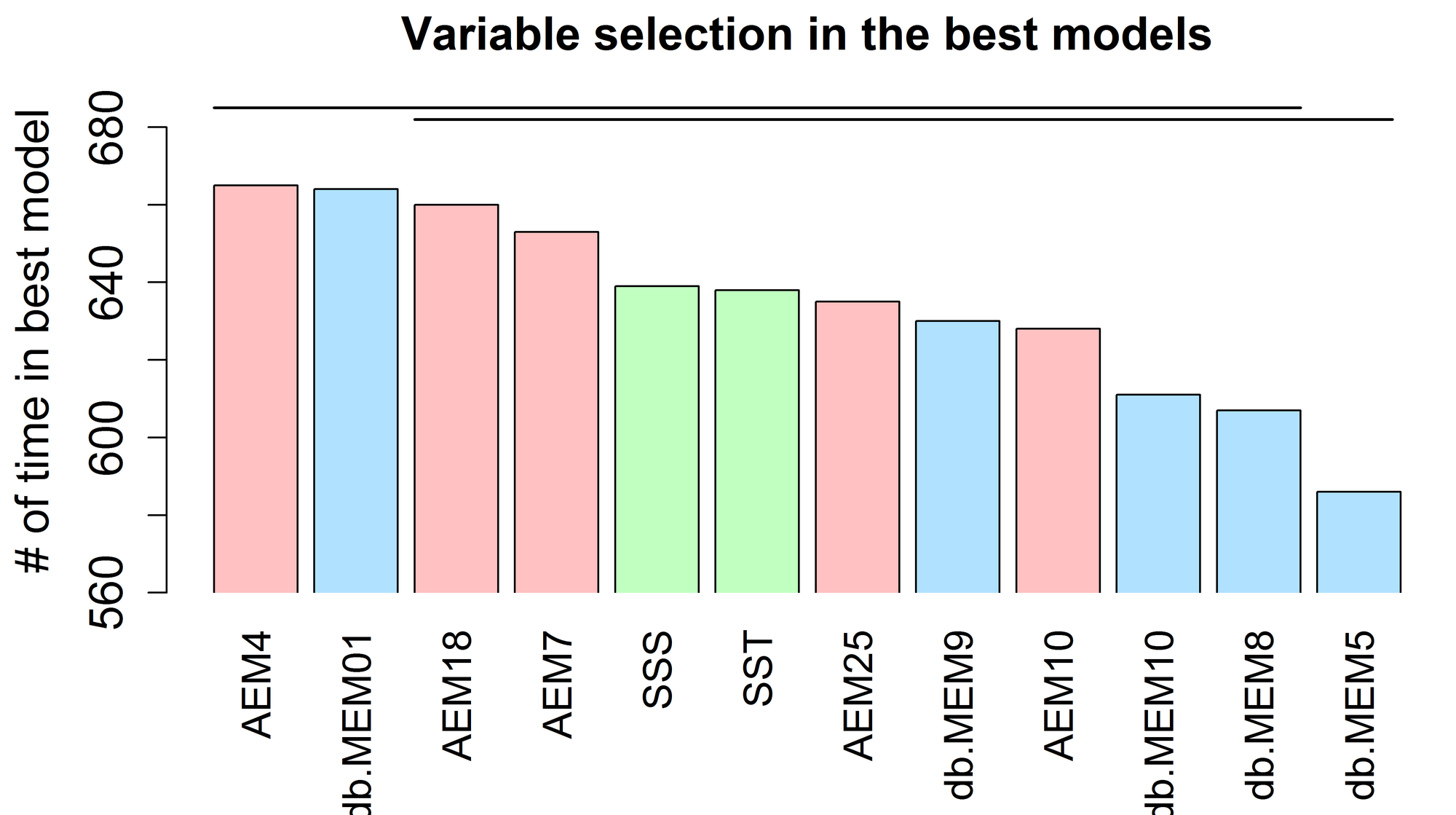


**Figure S5.**  Number of time (i.e. number of SNPs) that each variable has been selected in the best model. The two environmental variables (mean Sea Surface Temperature – SST and mean Sea Surface Salinity – SSS) are represented in green, the five geographic isolation vectors (db-MEMs), in blue and the five larval dispersal vectors (AEMs) in red. Horizontal segments above the graphs represent the results of the Dunn’s post-hoc test. A segment shows a group of variables whose differences in number of selection are not significant among them, but are significant with all other explanatory variables.

**Supporting Tables**

**Table S1**. Ecoregion, description, site number, location, number of adults of *Mullus surmuletus* sampled (*n*) and expected heterozygosity (H_S_) of the 47 sampling sites used in the analyses.

| **Ecoregion** | **Site** | **Site #** | **Country** | **Latitude** | **Longitude** | ***n*** | **Hs** |
| --- | --- | --- | --- | --- | --- | --- | --- |
| Atlantic | Gibraltar | 20 | Spain | 36.0155 | -5.2157 | 10 | 0.27 |
| Alboran Sea | Santa pola | 3 | Spain | 38.1684 | -0.1918 | 9 | 0.261 |
|  | Marbella | 4 | Spain | 36.5990 | -4.0531 | 9 | 0.263 |
|  | Aguilas | 5 | Spain | 36.8924 | -1.9338 | 9 | 0.268 |
| Western Mediterranean | Denia | 45 | Spain | 39.8159 | 0.3926 | 10 | 0.259 |
|  | Torredembara | 46 | Spain | 40.9500 | 2.0600 | 10 | 0.26 |
|  | Menorca | 100 | Spain (Balearic) | 39.8701 | 3.9435 | 9 | 0.258 |
|  | East Mallorca | 24 | Spain (Balearic) | 39.4800 | 3.5300 | 10 | 0.258 |
|  | Ibiza | 68 | Spain (Balearic) | 38.8311 | 1.3928 | 9 | 0.264 |
|  | West Mallorca | 99 | Spain (Balearic) | 39.4056 | 2.5751 | 9 | 0.271 |
|  | Marseille | 48 | France | 43.1260 | 4.7681 | 10 | 0.248 |
|  | Bonifacio | 14 | France (Corsica) | 41.2288 | 9.5491 | 10 | 0.26 |
|  | Cala Gonone | 16 | Italy (Sardinia) | 40.3453 | 9.8254 | 10 | 0.261 |
|  | North Corsica | 19_27 | France (Corsica) | 42.9300 | 9.1700 | 17 | 0.268 |
|  | East Sardinia | 13_15 | Italy (Sardinia) | 41.0100 | 8.1600 | 17 | 0.268 |
|  | Cagliari | 18 | Italy (Sardinia) | 39.1072 | 9.3990 | 9 | 0.26 |
|  | Tropea | 51 | Italy | 39.3350 | 15.7479 | 9 | 0.255 |
|  | Capri | 52_53 | Italy | 41.2109 | 13.0726 | 16 | 0.261 |
|  | Annaba | 88 | Algeria | 37.2780 | 7.2594 | 10 | 0.264 |
|  | Bizerte | 89 | Tunisia | 37.3368 | 9.0391 | 10 | 0.257 |
| Gulf of Sidra | Tunis | 90_92 | Tunisia | 35.6302 | 11.1504 | 17 | 0.267 |
|  | Zarsis | 91 | Tunisia | 33.3467 | 12.1275 | 10 | 0.258 |
| Ionian Sea | Crotone | 25_84 | Italy | 38.5500 | 16.7700 | 11 | 0.256 |
|  | Gallipoli | 85 | Italy | 40.0263 | 17.2276 | 9 | 0.248 |
|  | South Sicily | 12_87 | Italy (Sicily) | 36.8100 | 15.2600 | 15 | 0.261 |
|  | West Sicily | 11_86 | Italy (Sicily) | 37.7800 | 12.3200 | 18 | 0.26 |
|  | Kefalonia | 32_43 | Greece | 36.9400 | 21.5300 | 11 | 0.262 |
| Adriatic | Monfalcone | 80_82 | Italy | 43.6570 | 14.6014 | 14 | 0.261 |
|  | Monopoli | 83 | Italy | 40.6367 | 18.0343 | 10 | 0.252 |
| Aegean Sea | North Crete | 29_96 | Greece | 35.2844 | 25.7697 | 17 | 0.266 |
|  | Keramoti | 30 | Greece | 40.6046 | 24.4465 | 9 | 0.248 |
|  | Pereas | 34 | Greece | 40.0470 | 22.8903 | 10 | 0.253 |
|  | Naxos | 35 | Greece | 36.7800 | 25.5800 | 9 | 0.255 |
|  | Galatas | 36_8 | Greece | 37.8249 | 24.1212 | 12 | 0.263 |
|  | Sifnos | 37 | Greece | 37.2232 | 24.6743 | 10 | 0.253 |
|  | Skyros | 38_39 | Greece | 39.1457 | 23.9894 | 11 | 0.258 |
|  | Karpathos | 40_41 | Greece | 36.3271 | 27.4313 | 14 | 0.257 |
|  | Pyrgadikia | 9 | Greece | 40.0641 | 23.5063 | 18 | 0.245 |
|  | South Crete | 95 | Greece | 34.7800 | 25.2167 | 10 | 0.258 |
|  | Cesme | 6 | Turkey | 38.5700 | 26.1900 | 9 | 0.25 |
|  | Assos | 33_69 | Turkey | 39.6117 | 26.0826 | 13 | 0.258 |
| Levantine Sea | Gazipasha | 55_56 | Turkey | 35.9400 | 33.2368 | 18 | 0.259 |
|  | Samandag | 72 | Turkey | 36.2235 | 35.6724 | 10 | 0.256 |
|  | Kumyali | 10 | Cyprus | 35.3433 | 34.4483 | 10 | 0.257 |
|  | West Cyprus | 93_94 | Cyprus | 34.7800 | 32.3000 | 16 | 0.263 |
|  | Acre | 73 | Israel | 33.7650 | 35.1200 | 9 | 0.258 |
|  | Jaffa | 74 | Israel | 31.6697 | 34.0439 | 9 | 0.247 |

**Table S2**. Number of raw reads and filtering data for each sequenced library.

| **Library name** | **# raw sequences** | **# filtered sequences** | **%**  **filtered** |
| --- | --- | --- | --- |
| raw_C6JATANXX_1_fastq.txt | 293626046 | 152365277 | 51.89 |
| raw_C6JATANXX_2_fastq.txt | 265698080 | 250811438 | 94.40 |
| raw_C6JATANXX_3_fastq.txt | 272001986 | 254706376 | 93.64 |
| raw_C6JATANXX_4_fastq.txt | 264688559 | 206020993 | 77.84 |
| raw_C6JATANXX_5_fastq.txt | 292012357 | 271356748 | 92.93 |
| raw_C6JATANXX_6_fastq.txt | 279153586 | 255075707 | 91.37 |
|  |  | Average = | 83.68 |
